# The characteristics and perioperative outcomes of children with orofacial clefts managed at an academic hospital in Johannesburg, South Africa

**DOI:** 10.1186/s12887-022-03267-5

**Published:** 2022-04-19

**Authors:** Prosperity A. Sithole, Palesa Motshabi-Chakane, Michel K. Muteba

**Affiliations:** Department of Anaesthesiology, University of the Witwatersrand, Johannesburg, South Africa

**Keywords:** Orofacial clefts, Syndromic clefts, Concomitant congenital anomalies

## Abstract

**Background:**

Orofacial clefts (OFCs) are the commonest congenital anomalies of the head and neck. Their aetiology is multifactorial, and prevalence has a geographical variation. This study sought to describe OFC cases that presented for surgery.

**Objectives:**

The study aimed to describe the preoperative characteristics, concomitant congenital anomalies and perioperative outcomes of children presenting for cleft repair surgery over a 5-year period at Charlotte Maxeke Johannesburg Academic Hospital (CMJAH).

**Methods:**

A retrospective descriptive record review for children under the age of 14 years who presented for cleft repair surgery at Charlotte Maxeke Johannesburg Academic Hospital (CMJAH) during a 5-year period, from 1 January 2014 to 31 December 2018. Descriptive and comparative statistics were used to report the results.

**Results:**

A total of 175 records were included in the study. The median (IQR) age was 11 (6—27) months, with a predominance of males 98 (56%). Most of the children had cleft lip and palate (CLP) 71(41%). The prevalence of concomitant congenital anomalies was 22%, emanating mostly from head and neck congenital anomalies. Nine syndromes were identified in 15 children with syndromic clefts. Twenty-nine percent of children were underweight for age. There were 25 anaesthetic related complications, commonly airway related. Six children with complex multiple congenital anomalies were admitted in the intensive care unit postoperatively. No mortalities were recorded.

**Conclusion:**

Majority of children with orofacial clefts underwent cleft repair surgery without serious complications and intensive care unit admission. Only six children were diagnosed with significant anomalies needing intensive care management.

## Introduction

Orofacial clefts (OFCs) are a spectrum of congenital defects caused by failure of fusion of craniofacial processes that form the primary and secondary palates. They are classified according to the presenting anatomical defect, as cleft lip (CL), cleft lip with cleft palate (CLP) or cleft palate alone (CP) [1]. There is a worldwide occurrence with geographical variations in incidence. The highest incidence was reported in Japan with 2: 1000 [2]. In Africa a low incidence of 0.4:1000 was reported in Nigeria [3] and 0.3:1000 in South Africa [4].

Prevalence of concomitant congenital anomalies in children with orofacial clefts has been reported to be between 4.3% to 63.4% [5, 6]. The central nervous system, head and neck, cardiovascular, musculoskeletal, and urogenital system are commonly affected [2, 5, 6]. A South African study found a prevalence of concomitant cardiac anomalies in children with orofacial clefts of 30.7% in one hospital [7]. There are reportedly over 150 syndromes associated with orofacial clefts [7, 8]. Respiratory infections, malnutrition and anaemia are common medical co-morbidities [6].

Genetic and environmental factors are associated with the aetiology of orofacial clefts. Advanced maternal age at conception, antenatal smoking, alcoholism, vitamin deficiencies, maternal intake of anticonvulsants [2] and poor social circumstances were reported risks [2, 9]. Kawalec et al. [10] list associated environmental risk factors including pesticides, organic solvents, electromagnetic radiation, copper, biocides and aliphatic aldehydes and acids.

Early repair of orofacial clefts was recommended to achieve better facial cosmesis, normal feeding, dental and speech development [4]. Anaesthetic techniques described were local anaesthesia, general anaesthesia, and a combination of both, which has been recommended [11]. Anaesthetic complications were associated with age of children, anaesthetic technique, patient’s medical comorbidities and concomitant congenital anomalies. Neonates and infants were at greater risk [12, 13]. General anaesthesia had more complications than local anaesthesia [13]. Children with concomitant cardiac anomalies [7], and those with identified syndromes like Pierre Robin, [13] had more anaesthetic complications.

In South Africa, data on orofacial clefts has mostly been on epidemiological studies, clinical profiles of children with orofacial clefts, and caregiver’s perspectives. One study described prevalence of concomitant cardiac anomalies and possible anaesthetic implications [7]. We have not found data on aspects of concomitant congenital anomalies and their effect on clinical outcomes of surgery under anaesthesia. This study sought to describe the preoperative characteristics, concomitant congenital anomalies and perioperative outcomes of children presenting for cleft repair surgery over a 5-year period at Charlotte Maxeke Johannesburg Academic Hospital (CMJAH).

## Materials and methods

This was a retrospective descriptive record review of children who had cleft repair surgery at CMJAH between 1 January 2014 and 31 December 2018. The study included children under the age of 14 who presented for orofacial cleft repair surgery. Children older than 14 and those whose records were missing were excluded from the study. A complete enumeration survey was performed.

### Ethical considerations

The Human Research Ethics committee (HREC) of the University of the Witwatersrand in Johannesburg provided ethical approval for this study. This was a retrospective record review, so the need for written consent was waived by the Human Research Ethics Committee (HREC) of the University of the Witwatersrand in Johannesburg. The clearance certificate number was M200206.

All methods were carried out in accordance with the relevant guidelines and regulations in the Declaration of Helsinki [14]. Permission to use children’s records was granted by the hospital chief executive officer, heads of departments of theatre, anaesthetics, plastic surgery, and paediatric cardiology. There was no physical contact with children and data were deidentified. No records were taken off the Hospital premises.

#### Data management

Data for this study was collected and managed using the Research Electronic Data Capture (REDCap) [15, 16], hosted by University of the Witwatersrand. REDCap is a secure, web-based software platform designed to support data capture for research studies, providing: 1) an intuitive interface for validated data capture; 2) audit trails for tracking data manipulation and export procedures; 3) automated export procedures for seamless data downloads to common statistical packages; and 4) procedures for data integration and interoperability with external sources.

The main sources of data were anaesthetic charts and the children’s clinical notes saved on the Hospital electronic database. Data collected included sociodemographic variables (gender, age, race/ nationality, and employment status of parents). Prenatal and birth history data were recorded. Risk factors associated with development of orofacial clefts such as maternal age, social habits and family history of clefts were also recorded, where available. Preoperative diagnosis of associated congenital and medical conditions, intraoperative events including monitoring, anaesthetic techniques and complications were recorded. Postoperative outcome data (complications, post-operative destination and hospital stay) were captured.

For the purposes of this study, the orofacial clefts were divided into isolated cleft lip (CL), cleft lip and palate (CLP) and isolated cleft palate (CP) [6]. We did not look at atypical clefts separately in this study. Orofacial clefts associated with a syndrome were classified as syndromic orofacial clefts. Clefts associated with multiple concomitant congenital anomalies which did not fit into a syndrome were classified as multiple congenital anomalies of unknown origin (MCAs) [6].

#### Statistical analysis

Socio-demographic data, cleft classes, concomitant congenital anomalies, and perioperative events were described using proportions and frequencies for categorical data, means (SD) for normally distributed continuous data and median (IQR) for non-normally distributed continuous data. Stata 16.1 (StataCorp, USA) was also used for comparative analysis [17]. Generalised linear models for binomial analysis were used for age and weight. Fishers exact test was used for comparisons with small samples.

## Results

**Fig. 1:**
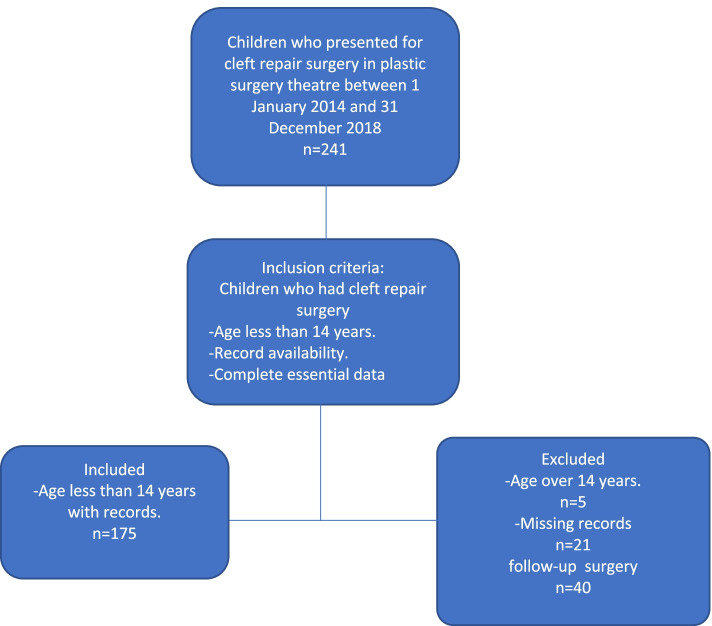
Flow diagram of the participant selection process

There were 175 eligible records of children who underwent cleft repair surgery during the study period**.** The participant selection process is shown in the diagram (Fig. 1) below.

The median (IQR) age at presentation was 11 (6—27) months. The median (IQR) weight was 8 (6.9—10.1) kg. The weight scale for age showed that 51 (30%) were malnourished. Perinatal history revealed that 131 (75%) were delivered vaginally, 25 (15%) by caesarean section for obstetric indications, and 1(0.6%) had vacuum-assisted delivery. The majority were male 98 (56%), and Black African 133 (76%).

Table 1 shows risk factors associated with development of orofacial clefts in this study. Maternal HIV infections during pregnancy were reported in 29 (17%) cases. In this group, one had rubella, one tuberculosis, and another had a malignancy for which she received chemotherapy during the pregnancy. Only 56 (32%) had taken vitamin and mineral supplements during pregnancy.

**Table 1:** Important risk factors associated with development of orofacial clefts

Risk factor	n (%)
Family history and genetic factors	18(10)
Alcohol use during pregnancy	4(2)
Tobacco use during pregnancy	9(5)
Medications taken during pregnancy	1(0.6)
Maternal infections during pregnancy	29(17)
Maternal age over 35 years	16(9)
Low socioeconomic status	62(35)
Use of illicit drugs during pregnancy	2(2)
Antenatal vitamin and mineral supplements	56(32)

Preoperatively, the children were classified using the American Society of Anaesthesiologists (ASA) classification [18]. Most were ASA 1, as reported by the attending anaesthetist, [118 (67%)]. The prevalence of concomitant congenital anomalies in this study was 38 (22%). Of these, 28 (82%) had obvious dysmorphic features. Syndromes were identified in 15 children (40%), whilst 23 (60%) had MCAs (Table 2). One patient who presented with craniosynostosis had a craniotomy prior to cleft surgery due to raised intracranial pressure. Preoperative echocardiography was performed for 10 children on clinical suspicion of presence of congenital cardiac disease and only 2 (20%) were confirmed to have structural heart abnormalities [patent ductus arteriosus (PDA) and Tetralogy of Fallot (TOF)]. The patient with a TOF needed cardiac surgery prior to their cleft palate repair surgery.

**Table 2:** Identified syndromes among 38 children with concomitant anomalies

Syndrome (*N* = 38)	n (%)
Amniotic band syndrome	5(13)
Pierre Robin Sequence	3(8)
Goldenhar syndrome	1(3)
Apert syndrome	1(3)
Wolf-Hirschhorn syndrome	1(3)
Ectrodactyly ectodermal dysplasia CLP syndrome (EEC syndrome)	1(3)
Arthrogryposis multiplex congenita	1(3)
Moebius syndrome	1(3)
Congenital rubella syndrome	1(3)

Table 3 shows a descriptive list of the diagnosed concomitant congenital anomalies. The most common early cleft-related complication in this study was feeding difficulties and malnutrition (underweight for age) 51 (29%), with 3 children requiring percutaneous endoscopic gastrostomy feeding tubes. Recurrent upper respiratory tract infections occurred in 9 (5%) children. Late complications were persistent speech challenges 16 (9%), neurodevelopmental delay because of malnutrition (underweight) and failure to thrive 13 (7%), and persistent nasal regurgitation of meals 4 (2%).

**Table 3:** Descriptive list of concomitant congenital anomalies by system

System	Abnormality
Head and neck/ neurological	Head: Microcephaly, craniosynostosis, plagiocephaly, scaphocephaly, frontal bossing, midface hypoplasia, micrognathia, mental retardation, short neck, flat nasal bridge, glosso-ptosis Neurological: cranial nerve palsies, cerebral palsy Eyes: cataract, amniotic bands, coloboma, anophthalmia, nystagmus, ptosis Ears: low-set, absent external auditory meatus, complete hearing loss
Musculoskeletal	Muscular dystrophy, clubfoot, cleft hands and feet, syndactyly, limb contractures, scoliosis, kyphosis, hypotonia, amniotic bands, missing phalanges, claw hands and feet
Cardiovascular	Patent Ductus Arteriosus, Tetralogy of Fallot
Urogenital	Absent kidney, ectopic kidney, undescended testicles
Respiratory system	Pectus excavatum, hypoplastic lung, rib abnormalities
Gastrointestinal	Umbilical hernia

Anaesthetic technique was general anaesthesia with endotracheal intubation for all children. Co-induction was done with sevoflurane, propofol and fentanyl for all except one. The majority were intubated with a direct laryngoscope, with 138 (79%) successful first attempts, 18 (10%) were difficult intubations (3 or more attempts) and 4 (2%) had planned video laryngoscopy because they were already predicted difficult airways. Anaesthesia was maintained with sevoflurane. A single child with congenital myopathy received trigger-free anaesthesia, with propofol and remifentanil total intravenous anaesthesia. Analgesia was multimodal with intravenous agents such as acetaminophen, ketamine, fentanyl, and magnesium sulphate. Some received rectal acetaminophen. In addition, 58 (33%) had local anaesthetic (xylocaine) infiltrated by the surgeons, whilst 17 (10%) had peripheral nerve blocks with bupivacaine.

Predominantly, unilateral cleft lip repairs were performed [70 (40%)], followed by first-stage palate repair [64 (37%)], then bilateral cleft lip repair [15 (9%)]. The rest were cleft nose repair [11 (6%)], second stage palate repair [10 (6%)], oronasal fistula repair [2 (1%)], and lip and revision procedures [2 (1%)].

Postoperative complications occurred in 11 (6%) children, with a total of 25 events (Table 4). One patient bled in recovery, requiring reintubation and surgical relook. The early postoperative anaesthetic complications were respiratory 10 (6%), with one requiring reintubation, and cardiovascular collapse [1 (0.6%)]. Intensive care admission was required for 6 (3%) of the children, and all had complex multiple congenital anomalies.

**Table 4:** Occurrence of anaesthetic complications

Complication	Total (*N* = 175) n (%)
Accidental extubation	1 (0.6%)
Desaturation	6 (3%)
Hypercarbia	1 (0.6%)
Hyperthermia	1 (0.6%)
Laryngospasm	9 (5%)
Bronchospasm	4 (2%)
Aspiration	1 (0.6%)
Bradycardia	1 (0.6%)
Delayed emergence	1 (0.6%)

There were no significant differences between children who had isolated clefts and those who had concomitant congenital anomalies when age and weight at presentation were compared, (Table 5). There were also no differences in occurrence of laryngospasm, desaturation nor postoperative respiratory complications. There was a significant difference in duration of hospital stay after surgery *p* = 0.017 and ICU stay *p* < 0.001.

**Table 5:** Comparisons of complications between those with and without concomitant congenital anomalies

Variable		Isolated cleft lip and/palate *N* = 137	Cleft and concomitant anomalies *N* = 38	*P* value
**Age**		10 (5–30)	14 (6–19)	0.286
**Weight**		8 (7–9)	8 (7–9)	0.207
**Postoperative respiratory complications**		9 (7%)	1 (3%)	0.692
**Laryngospasm**		7 (5%)	2 (5%)	1.00
**Desaturation**		5 (4%)	1 (3%)	1.00
**Postoperative ICU admission**		0 (0%)	6 (16%)	< 0.001
**Postoperative hospital stay**	0 -5 days	115 (98%)	24 (80%)	0.017
	≥ 6 days	3 (2%)	6 (20%)	
	*Missing*	*19*	*8*	

## Discussion

Children presented with a median age of 11 months and median weight of 8 kg. The youngest patient was 3 months old (12 weeks). Half of the children presented in a malnourished state. Anaemia was excluded clinically on examination, and therefore only two children had their haemoglobin investigated before surgery. A previous study has reported respiratory infections, malnutrition, and anaemia to be common among children with OFC [6]. The decision on timing of surgery was made using Kilner’s rule of 10. The rule states that the patient must be at least 10 weeks old, weighing at least 10 pounds (4.5 kg), with a haemoglobin of 10 g/dl [19]. We found only two children weighing less than 4.5 kg. Our children were predominantly male.

Previous studies have shown a predominance of males [1, 6, 10, 20], albeit that a multi-centre epidemiological study in South Africa had shown a higher proportion of females (53%) [21]. We postulate that children were malnourished due to difficulties in feeding related to the cleft pathology.

Although the aetiology of orofacial cleft is mostly not known [19, 22], multiple factors that have been previously studied and classified as genetic or environmental, were identified in this study. Low socioeconomic status, was measured by the number of unemployed parents who relied on government grants for income, Advanced maternal age, which was also found in our population, described as maternal age over 35 years at the time of conception [10], has been a point of conflicting results. Studies by Jamilian et al. [23] and Paranaiba et al. [9], showed no relationship. On the contrary, a Danish study concluded that both advanced paternal and maternal age were associated with orofacial clefts [24].

Use of tobacco and alcohol during pregnancy was shown to be strongly association with orofacial clefts in previous studies [1, 2, 5, 10]. Studies by Altunhan et al. [5], and Paranaiba et al. [9] on the contrary found no significant relationship between environmental factors and orofacial clefts. Use of illicit drugs and exposure to chemotherapeutic drugs have also been described [10].

Maternal infections have also been listed as a possible aetiological factor for orofacial clefts [2, 10]. All the mothers who had documented infections antenatally had (Human Immune Deficiency Virus) HIV infection. There have not been many studies in this area, although a previous study concluded that there might be a possible role of antiretroviral drugs in development of orofacial clefts [25]. The regimens implicated were Tenofovir/Lamivudine/Efavirenz regimen and Zidovudine/Lamivudine/Nevirapine [25]. Another study, concluded that children exposed to HIV in utero who had orofacial clefts, suffered from oropharyngeal dysphagia, as opposed to non-exposed children, concluding that HIV had a neurological impact on exposed children [26]. Other environmental factors that have been described are poor maternal nutrition, vitamin and mineral deficiencies [2, 10]. We did not investigate these but could infer a relationship through data on socioeconomics.

Over 150 syndromes are known to be associated with orofacial clefts whilst an estimated 70% of orofacial clefts are non-syndromic [8]. This study identified 9 syndromes in 15 of the children. A study in Burkina Faso identified 5 syndromes in children with CLP [6], whilst one performed in Central South Africa identified 4 syndromes, 2 in CL and 2 in CP [7]. In this study, 6 (40%) of syndromic clefts had cleft palate, 7 (47%) had cleft lip and palate whilst 2 (13%) had cleft lip.

According to a review by Law and De Klerk, the best mode of anaesthesia for cleft lip surgery is general anaesthesia and endotracheal intubation, in the younger children [27]. For cleft palate surgery, they recommended general anaesthesia with endotracheal intubation [27]. In this study, general anaesthesia with inhalational agents was performed for all children except one myopathic child who had total intravenous anaesthesia. Maxillary nerve blocks for cleft palate surgery have been shown to provide good intraoperative and postoperative analgesia for cleft surgery children [11]. It also reduces intraoperative opioid use and reduces the stress response to surgery [11]. For cleft lip surgery, infraorbital nerve blocks were performed in this study.

General anaesthesia is associated with complications. In a study done in a resource-limited setting, local anaesthesia reduced complications [20]. The complications encountered in our study have been described in other studies, [12, 28, 29] and they were successfully managed. In Burkina Faso no perioperative complications were reported and it was concluded that surgery for orofacial clefts was safe [30]. Akitoye et al. [20], also confirmed safety of general anaesthesia in their study conducted in a resource limited environment. We reported no perioperative mortalities in this study. No studies have reported perioperative mortality during cleft repair surgery.

### Limitations

The study depended solely on the preservation and accuracy of the children’s hospital records. This was a single-hospital study and therefore the findings should be interpreted with caution, they cannot be applied to the general population.

## Conclusion

Children with orofacial clefts need to be thoroughly preoperatively. Some concomitant congenital anomalies may need to be surgically corrected before the orofacial clefts can be repaired. The prevalence of clinically suspected concomitant cardiac anomalies in children with orofacial clefts was low in this study with very few needing preoperative echocardiogram. Overall, children with OFCs have been managed safely at CMJAH with no perioperative mortalities recorded.

## Data Availability

The datasets used during the current study are available from the corresponding author on reasonable request.

## Abbreviations

ASA: American Society of Anaesthesiologists
CL: Cleft lip
CLP: Cleft lip and palate
CMJAH: Charlotte Maxeke Johannesburg Academic Hospital
CP: Cleft palate
HB: Haemoglobin
HIV: Human Immune deficiency virus
IQR: Interquartile range
MCA: Multiple congenital anomalies of unknown origin
OFCs: Orofacial clefts
PDA: Patent ductus arteriosus
TOF: Tetralogy of Fallot
